# Saroglitazar is noninferior to fenofibrate in reducing triglyceride levels in hypertriglyceridemic patients in a randomized clinical trial

**DOI:** 10.1016/j.jlr.2022.100233

**Published:** 2022-05-21

**Authors:** Rene Rodriguez-Gutierrez, Jose Gerardo González, Deven Parmar, Farheen. Shaikh, Pio Cruz-López

**Affiliations:** 1 UANL,Monterrey, México and Mayo Clinic, Rochester, MN, USA, Endocrinology, Monterrey, Mexico; 2 UANL,Monterrey, México. Facultad de Medicina y Hospital Universitario "Dr. José Eleuterio González" Endocrinology, Monterrey, Mexico; 3 ZydusTherapeutics Inc., Clinical Research and Development, Pennington, New Jersey, USA; 4 Zydus Worldwide DMCC, Clinical Research and Development, Dubai, United Arab Emirates; 5 Avant Sante Research Center SA de CV., Clinical Development, Monterrey, Mexico

**Keywords:** atherosclerosis, lipids, cholesterol/metabolism, obesity, PPARs, dyslipidemias, LDL/metabolism, triglycerides, lipolysis, fatty acid metabolism, AE, adverse event, CAP, continuation attenuation parameter, FPG, fasting plasma glucose, HbA1c, hemoglobin A1c, LS, least square, PP, per-protocol, TC, total cholesterol, TG, triglyceride

## Abstract

Saroglitazar, being a dual PPAR-α/γ agonist, has shown beneficial effect in diabetic dyslipidemia and hypertriglyceridemia. Fibrates are commonly used to treat severe hypertriglyceridemia. However, the effect of saroglitazar in patients with moderate to severe hypertriglyceridemia was not evaluated. We conducted a study to compare the efficacy and safety of saroglitazar (4 mg) with fenofibrate (160 mg) in patients with moderate to severe hypertriglyceridemia. This was a multicenter, randomized, double-blinded, double-dummy, active-control, and noninferiority trial in adult patients with fasting triglyceride (TG) levels of 500–1,500 mg/dl. The patients were randomized in a 1:1 ratio to receive daily dose of saroglitazar or fenofibrate for 12 weeks. The primary efficacy end point was the percent change in TG levels at week 12 relative to baseline. The study comprised of 41 patients in the saroglitazar group and 41 patients in the fenofibrate group. We found that the percent reduction from baseline in TG levels at week 12 was significantly higher in the saroglitazar group (least square mean = −55.3%; SE = 4.9) compared with the fenofibrate group (least square mean = −41.1%; SE = 4.9; *P* = 0.048). Overall, 37 treatment-emergent adverse events (AEs) were reported in 24 patients (saroglitazar: 13; fenofibrate: 11). No serious AEs were reported, and no patient discontinued the study because of AEs. We conclude that saroglitazar (4 mg) is noninferior to fenofibrate (160 mg) in reducing TG levels after 12 weeks of treatment, was safe, and well tolerated.

Hypertriglyceridemia is one of the most common lipid abnormalities characterized by overproduction and impaired clearance of VLDL-C and chylomicrons leading to its accumulation in the circulation (1). Severe hypertriglyceridemia is associated with increased risk of cardiovascular events and acute pancreatitis (2). Cardiovascular risk can be reduced by controlling dyslipidemia, blood pressure, body weight, and hypertriglyceridemia (3).

Lifestyle and dietary modifications are the first-line treatment for hyperglyceridemia (4). Statins, fibrates, niacin, and n-3 fatty acids are clinically available for treatment for hyperglyceridemia (5). Fibrates, including fenofibrate, which activate PPARα, can improve triglyceride (TG) levels by 25–50% and HDL-C levels by 5–20% (6, 7, 8). However, fibrates have been associated with increased risk of liver damage and increased levels of serum creatinine (5, 9).

Several research groups have attempted to develop dual PPAR-α/γ agonist, considering role of PPARα agonist action on improvement of lipid parameters and PPARγ agonist action on improvement of insulin sensitivity and glycemic control (10).

Saroglitazar magnesium (saroglitazar) is a novel predominately PPARα and moderate PPARγ agonist. The PPARα activation by saroglitazar increases the hepatic oxidation of fatty acids, thus reducing the synthesis and secretion of TGs. In addition, it activates LPL, which increased lipolysis and eliminates TG-rich particles from plasma and also reduces production of apolipoprotein C-III (an inhibitor of LPL activity). It reduces plasma LDL-C and increases synthesis of apolipoproteins, A-I, A-II, and HDL-C. Saroglitazar increases the expression of numerous PPARγ-responsive genes including insulin-responsive genes, which controls the production, transport, and utilization of glucose. This enhances carbohydrate and lipid metabolism, including adiponectin, adipocyte fatty acid-binding protein (aP2), LPL, fatty acid transport protein, and fatty acid translocase (CD36) by increasing the expression of their respective genes. Preclinical and clinical studies have shown superior or equivalent safety and efficacy profile of saroglitazar compared with marketed fibrates or thiazolidinediones (11).

Previous clinical studies showed approximately 45% reduction in TG level after 12 weeks of saroglitazar 4 mg treatment in patients having mild hypertriglyceridemia (TG level between 200 mg/dl and 500 mg/dl) (12, 13). This study aims to establish whether treatment with saroglitazar for 12 weeks is noninferior to fenofibrate in patients with moderate to severe dyslipidemia defined as fasting TG level of 500–1,500 mg/dl.

## Materials and methods

### Study design

This was a multicenter, randomized, double-blind, double-dummy, and active-controlled study to evaluate the safety and efficacy of saroglitazar compared with fenofibrate in patients with moderate to severe hypertriglyceridemia defined as fasting TG level of 500–1,500 mg/dl. Ninety-four eligible patients at 10 participating medical centers in Mexico were enrolled between October 2017 and January 2020. The study protocol was reviewed and approved by the institutional review board at each participating medical center. The study was conducted in accordance with the International Conference on Harmonization—Good Clinical Practice guidelines and the ethical principles of the Declaration of Helsinki.

### Participants

Patients 18 years and older at the time of screening with key entry criterion as fasting TG level of ≥500 mg/dl at the screening visit and an average fasting TG level of ≥500 mg/dl to ≤1,500 mg/dl at the two qualification visits prior to randomization were enrolled. Patients with history of pancreatitis and weight gain or weight loss of >5% within 6 months of the initial screening visit were excluded from the study. All patients provided written informed consent prior to the study participation.

### Randomization and masking

Eligible patients were randomly assigned in a 1:1 ratio to receive daily dose of saroglitazar 4 mg or fenofibrate 160 mg for 12 weeks. A block randomization schedule was generated using SAS® software (version 9.4; SAS Institute, Inc, Cary, NC). Patients, investigators, clinical staff, pathologists, and individuals who were involved in the study-related activities were blinded to treatment assignment during the study conduct.

### Procedures

This trial was conducted over a period of up to 20 weeks, which included a 4- to 6-week run-in period, 2-week qualification period, and 12-week treatment period. A follow-up telephonic visit was performed to assess any adverse event (AE) within 72 h of last dose. Patients were followed every 4 weeks for clinical assessments. Liver stiffness measurement and continuation attenuation parameter (CAP) were performed by transient elastography using Fibroscan® at baseline and week 12.

Total cholesterol (TC) and TG levels were measured by enzymatic assay (Roche Modular), HDL-C and LDL-C levels were measured directly by colorimetric methods (Roche Modular), plasma insulin and C-peptide levels were measured using an electrochemiluminescence immunoassay (Roche Modular), apolipoprotein A-I and apolipoprotein B levels were quantified by immunonephelometric methods (Siemens GmbH, Germany), and adiponectin level was measured using ELISA kits (Biovendor-Laboratory Medicine, Inc). VLDL-C was estimated with TG level divided by five, and apolipoprotein C-III was measured using the turbidimetric immunoassay (Roche test).

### Outcomes

The primary efficacy end point was the percent change in TG level at week 12 relative to baseline. Secondary efficacy end points included the percent change from baseline in non-HDL-C, TC, LDL-C, HDL-C, VLDL-C, fasting plasma glucose (FPG), Apolipoprotein A, B, and C-III, glycosylated hemoglobin A1c (HbA1c), adiponectin, C-peptide, fasting insulin, liver function tests, liver stiffness, and CAP at all assessment time points. The safety was assessed by analysis of AEs, physical examination, vital signs, body weight, clinical laboratory evaluations, and 12-lead electrocardiogram.

### Statistical analysis

The primary and secondary efficacy end points were analyzed using the per-protocol (PP) population, which included all randomized patients who received at least one dose of study drug and completed the treatment phase with no protocol deviations that could affect the evaluation of efficacy assessments. All safety parameters were analyzed using safety population that included all randomized patients who received at least one dose of study drug. Baseline value for TG was defined as the average of three assessment values conducted at the qualification and baseline visits, whereas for all other parameters, it was defined as the last assessment prior to the administration of the first dose of study drug.

The primary efficacy end-point percent change in TG level at week 12 relative to baseline was analyzed using ANCOVA model using treatment as fixed effect and baseline value as a covariate. Least square (LS) means for each treatment group and associated SEs were provided. In addition, LS mean differences (fenofibrate minus saroglitazar magnesium) and 95% two-sided CIs for the treatment difference derived from the ANCOVA model were also provided. Noninferiority was concluded if the lower limit of 95% two-sided CI for the LS mean treatment group difference was greater than minus (–) 4%. The secondary efficacy end points were analyzed using an ANCOVA model similar to the primary efficacy end point. Subgroup analyses were conducted for participants with and without diabetes for the primary and secondary efficacy end points. AEs were summarized according to the Medical Dictionary for Regulatory Activities System Organ Class, the Medical Dictionary for Regulatory Activities preferred term, severity (as defined in the protocol), and causal relationship (as assessed by the individual investigators). All statistical testings were two-sided and performed at 5% level of significance. Statistical analyses were conducted using SAS® software.

## Results

### Study population

A total of 445 patients were screened from October 2017 and January 2020, and 94 eligible patients were randomized; 48 patients in the saroglitazar group and 46 patients in the fenofibrate group. Overall, 88 (93.6%) patients completed the study, and six (6.4%) patients discontinued from the study. The reasons for study discontinuation were withdrawal consent to participate the study in three patients, lost to follow-up in two patients, and lack of compliance in one patient. No patient discontinued the study because of AEs. Of the 88 patients completed the study, six patients had major protocol deviation and were excluded from the PP population. Hence, PP population consisted of 41 patients in the saroglitazar group and 41 patients in the fenofibrate group.

The demographic and baseline characteristics of the patients were generally comparable between the treatment groups (Table 1). Overall, the mean age of the study population was 49.6 (SD = 11.3) years, and 61.7% of the patients were males. Thirty-five (42.7%) patients reported history of diabetes, 26 (31.7%) patients reported history of hypertension, and 16 (19.5%) reported taking statin. The demographic and baseline characteristics of all participants as well as by diabetic group are presented in Table 1.

**Table 1:** Demographic and baseline characteristics

Parameters	Diabetes			No diabetes			All subjects		
	Saroglitazar 4 mg (*N* = 19)	Fenofibrate 160 mg (*N* = 16)	*P*	Saroglitazar 4 mg (*N* = 22)	Fenofibrate 160 mg (*N* = 25)	*P*	Saroglitazar 4 mg (*N* = 41)	Fenofibrate 160 mg (*N* = 41)	*P*
Age (in years)	54.4 (8.2)	51.5 (8.8)	0.325	47.8 (8.2)	48.2 (11.9)	0.888	50.8 (8.8)	49.5 (10.8)	0.539
Sex, *n* (%)									
Male	12 (63.2)	8 (50.0)	0.433	14 (63.6)	16 (64.0)	0.979	26 (63.4)	24 (58.5)	0.651
Female	7 (36.8)	8 (50.0)		8 (36.4)	9 (36.0)		15 (36.6)	17 (41.5)	
Body mass index	29.5 (3.8)	30.2 (4.7)	0.630	31.6 (7.1)	29.7 (5.4)	0.315	30.6 (5.8)	29.9 (5.1)	0.560
TG (mg/dl)	748.5 (293.2)	850.7 (398.1)	0.389	697.9 (202.3)	773.6 (211.5)	0.218	721.4 (246.6)	803.7 (296.2)	0.175
TC (mg/dl)	227.0 (47.8)	229.1 (81.6)	0.930	233.2 (45.0)	224.1 (68.9)	0.598	230.3 (45.8)	226.0 (73.1)	0.750
HDL (mg/dl)	33.1 (8.1)	34.1 (8.4)	0.723	33.2 (9.0)	31.9 (10.4)	0.652	33.1 (8.5)	32.7 (9.6)	0.843
LDL (mg/dl)	108.9 (42.5)	92.9 (38.9)	0.267	102.3 (44.7)	84.1 (50.9)	0.205	105.4 (43.3)	87.5 (46.3)	0.078
Non-HDL-C (mg/dl)	202.4 (70.8)	170.7 (53.5)	0.161	196.5 (47.0)	186.2 (63.5)	0.540	199.2 (58.5)	180.3 (59.6)	0.156
VLDL (mg/dl)	132.6 (102.2)	152.5 (147.3)	0.642	124.3 (67.4)	139.7 (75.0)	0.465	128.2 (84.3)	144.7 (107.5)	0.440
Apolipoprotein A1 (mg/dl)	134.6 (16.4)	138.3 (24.5)	0.601	134.6 (18.7)	130.5 (20.0)	0.472	134.6 (17.4)	133.5 (21.9)	0.807
Apolipoprotein B (mg/dl)	129.7 (22.4)	112.5 (25.1)	0.046	119.5 (22.3)	107.5 (29.6)	0.126	124.0 (22.6)	109.4 (27.7)	0.012
Apolipoprotein CIII (mg/l)	193.3 (43.0)	196.7 (44.6)	0.821	188.3 (47.4)	213.4 (46.3)	0.076	190.4 (45.0)	206.7 (45.8)	0.115
Adiponectin (μg/ml)	4.2 (2.5)	4.6 (2.0)	0.629	4.3 (2.1)	4.1 (1.5)	0.801	4.2 (2.3)	4.3 (1.7)	0.867
C-peptide (ng/ml)	4.0 (1.4)	4.0 (1.2)	0.961	4.0 (2.0)	4.3 (2.5)	0.644	4.0 (1.7)	4.2 (2.0)	0.644
FPG (mg/dl)	174.0 (64.8)	172.1 (62.1)	0.931	103.2 (20.7)	102.5 (23.0)	0.913	136.0 (58.2)	129.7 (54.3)	0.611
HbA1c (%)	7.7 (1.8)	8.0 (1.5)	0.555	5.8 (0.8)	5.4 (0.6)	0.096	6.7 (1.7)	6.4 (1.6)	0.537
Insulin (μUI/ml)	19.1 (10.0)	19.8 (7.5)	0.819	20.7 (18.9)	23.4 (26.2)	0.689	20.0 (15.2)	22.0 (20.9)	0.613
Creatinine (mg/dl)	0.9 (0.3)	0.8 (0.2)	0.043	0.9 (0.3)	0.9 (0.2)	0.989	0.9 (0.3)	0.8 (0.2)	0.199
Statin use, *n* (%)	5 (26.3)	5 (31.3)	0.748	3 (13.6)	3 (12.0)	0.867	8 (19.5)	8 (19.5)	1.000
History of hypertension, *n* (%)	6 (31.6)	6 (37.5)	0.713	7 (31.8)	7 (28.0)	0.775	13 (31.7)	13 (31.7)	1.000

All continuous values were presented with mean (SD).

### Effect on lipid parameters

**Figure 1:**
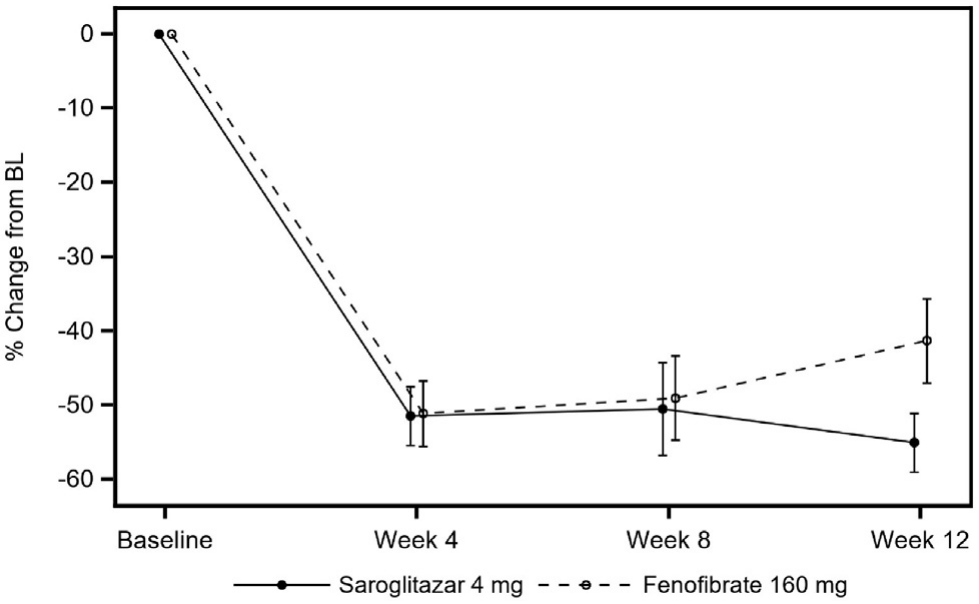
Percent change from baseline in TG level.

Treatment with both saroglitazar and fenofibrate resulted in rapid reduction of TG level at week 4 and sustained throughout the duration of treatment (Fig. 1). The mean percent reduction in TG level at week 12 relative to baseline was significantly higher in favor of saroglitazar 4 mg group (LS mean = −55.3%; SE = 4.9) compared with fenofibrate 160 mg group (LS mean = −41.1%; SE = 4.9) in the PP population (Table 2). The LS estimate of the mean difference between the treatment groups (fenofibrate 160 mg minus saroglitazar 4 mg) in percent change in TG level at week 12 was 14.1%, with the lower limit of the two-sided 95% CI being 0.14%, which is greater than the noninferiority margin of −4% and demonstrated the noninferiority of saroglitazar 4 mg to fenofibrate 160 mg at week 12 (Table 2). The result was consistent with the modified intent-to-treat population (results not provided). At week 12, a significantly higher number of patients in the saroglitazar group (85.4%) had TG level <500 mg/dl when compared with fenofibrate group (65.9%; *P* = 0.04, Chi-square test). Overall, 10 (24.4%) patients in the saroglitazar group and 5 (12.2%) patients in the fenofibrate group had normal TG levels defined as TG <150 mg/dl at week 12.

**Table 2:** Analysis of percent change from BL in TG (mg/dl) at week 12

	All subjects		Diabetes		No diabetes	
Visit	Saroglitazar 4 mg (*N* = 41)	Fenofibrate 160 mg (*N* = 41)	Saroglitazar 4 mg (*N* = 19)	Fenofibrate 160 mg (*N* = 16)	Saroglitazar 4 mg (*N* = 22)	Fenofibrate 160 mg (*N* = 25)
BL; LS mean (SE)	721.4 (42.6)	803.7 (42.6)	748.5 (79.1)	850.7 (86.2)	697.9 (44.2)	773.6 (41.5)
Week 12						
LS mean (SE)	314.5 (49.4)	474.0 (49.4)	371.6 (96.8)	536.2 (105.5)	265.2 (44.1)	434.2 (41.4)
LS mean % change from BL (SE)	−55.3 (4.9)	−41.1 (4.9)	−47.2 (7.6)	−43.3 (8.3)	−63.0 (6.4)	−39.1 (6.0)
Treatment difference (95% CI)	14.1 (0.1, 28.1)		3.9 (−19.1, 27.0)		23.9 (6.2, 41.6)	
*P*	0.048		0.732		0.009	

BL, baseline.

Note: Estimates were based on the ANCOVA model. BL TG value and treatment were included as factors in the model.

Overall, similar improvements in TC, non-HDL-C, VLDL-C, HDL-C, and apolipoprotein C-III levels were observed at week 12 relative to baseline in both the treatment groups (Table 3). At week 12, compared with fenofibrate, saroglitazar had no significant effect on the treatment differences in TC (2.9%; 95% CI: −6.1%, 11.8%), non-HDL-C (3.2%; 95% CI: −8.8%, 15.3%), VLDL-C (16.3%; 95% CI: −2.5%, 35.0%), HDL-C (−8.7%; 95% CI: −19.5%, 2.2%), or apolipoprotein C-III (1.1%; 95% CI: −14.7%, 16.8%) levels (Table 3).

**Table 3:** Analysis of percent change from BL in lipid and glucose control parameters at week 12

Parameter	Saroglitazar 4 mg (*N* = 41)	Fenofibrate 160 mg (*N* = 41)
Visit		
TC		
BL; LS mean (SE)	230.3 (9.53)	226.0 (9.53)
Week 12		
LS mean (SE)	203.2 (7.99)	203.4 (7.99)
LS mean % change from BL (SE)	−9.0 (3.18)	−6.1 (3.18)
Treatment difference (95% CI)	2.9 (−6.10, 11.83)	
*P*	0.527	
HDL		
BL; LS mean (SE)	33.1 (1.41)	32.7 (1.45)
Week 12		
LS mean (SE)	40.3 (1.71)	36.6 (1.76)
LS mean % change from BL (SE)	23.5 (3.78)	14.8 (3.92)
Treatment difference (95% CI)	−8.7 (−19.51, 2.18)	
*P*	0.116	
LDL		
BL; LS mean (SE)	105.4 (6.99)	87.5 (7.17)
Week 12		
LS mean (SE)	122.8 (5.55)	98.8 (5.69)
LS mean % change from BL (SE)	38.6 (7.82)	23.1 (8.13)
Treatment difference (95% CI)	−15.6 (−38.24, 7.12)	
*P*	0.176	
VLDL		
BL; LS mean (SE)	128.2 (15.08)	144.7 (15.08)
Week 12		
LS mean (SE)	62.5 (9.90)	94.4 (9.90)
LS mean % change from BL (SE)	−40.0 (6.65)	−23.7 (6.65)
Treatment difference (95% CI)	16.3 (−2.49, 35.01)	
*P*	0.088	
Non-HDL-C		
BL; LS mean (SE)	199.2 (9.22)	180.3 (9.45)
Week 12		
LS mean (SE)	162.9 (6.63)	159.4 (6.80)
LS mean % change from BL (SE)	−11.1 (4.16)	−7.8 (4.33)
Treatment difference (95% CI)	3.2 (−8.81, 15.28)	
*P*	0.594	
Apolipoprotein C-III		
BL; LS mean (SE)	189.75 (7.030)	210.04 (6.939)
Week 12		
LS mean (SE)	152.92 (10.664)	159.41 (10.664)
LS mean % change from BL (SE)	−19.58 (5.592)	−18.49 (5.519)
Treatment difference (95% CI)	1.09 (−14.68, 16.85)	
*P*	0.891	
Adiponectin		
BL; LS mean (SE)	4.23 (0.320)	4.30 (0.320)
Week 12		
LS mean (SE)	6.26 (0.460)	4.40 (0.454)
LS mean % change from BL (SE)	49.28 (7.184)	6.19 (6.997)
Treatment difference (95% CI)	−43.08 (−63.07, −23.10)	
*P*	<0.001	
C-peptide		
BL, LS mean (SE)	3.96 (0.293)	4.16 (0.293)
Week 12		
LS mean (SE)	3.42 (0.234)	3.70 (0.234)
LS mean % change from BL (SE)	−10.84 (4.042)	−6.12 (4.042)
Treatment difference (95% CI)	4.72 (−6.66, 16.11)	
*P*	0.411	
Creatinine		
BL; LS mean (SE)	0.89 (0.036)	0.82 (0.036)
Week 12		
LS mean (SE)	0.89 (0.046)	0.91 (0.046)
LS mean % change from BL (SE)	0.50 (3.097)	10.73 (3.097)
Treatment difference (95% CI)	10.24 (1.48, 19.00)	
*P*	0.023	
FPG		
BL; LS mean (SE)	136.0 (8.79)	129.7 (8.79)
Week 12		
LS mean (SE)	123.6 (8.76)	132.7 (8.76)
LS mean % change from BL (SE)	−6.0 (2.45)	1.9 (2.45)
Treatment difference (95% CI)	8.0 (1.08, 14.89)	
*P*	0.024	
HbA1c		
BL; LS mean (SE)	6.65 (0.259)	6.42 (0.259)
Week 12		
LS mean (SE)	6.56 (0.270)	6.73 (0.270)
LS mean % change from BL (SE)	−0.39 (1.423)	4.28 (1.423)
Treatment difference (95% CI)	4.67 (0.66, 8.68)	
*P*	0.023	
Insulin		
BL; LS mean (SE)	19.96 (2.857)	22.01 (2.857)
Week 12		
LS mean (SE)	16.88 (2.203)	17.99 (2.203)
LS mean % change from BL (SE)	−7.85 (8.668)	−5.12 (8.668)
Treatment difference (95% CI)	2.73 (−21.69, 27.15)	
*P*	0.825	

BL, baseline.

Note: Estimates were based on the ANCOVA model. BL values and treatment were included as factors in the model.

In this study, an increase in LDL-C levels at week 12 relative to baseline was observed in both the treatment groups. At week 12, a significant increase in adiponectin level relative to baseline was observed in favor of saroglitazar (LS mean = 49.3%; SE = 7.2) group compared with fenofibrate group (LS mean = 6.2%; SE = 7.0; *P* < 0.001).

### Effect on glucose control

A significant reduction in FPG at week 12 was observed in favor of saroglitazar group (−6.0%) when compared with an increase (1.9%; *P* = 0.024) in fenofibrate group (Table 3). A reduction was observed in insulin and C-peptide levels in both the treatment groups. But, the differences were not statistically significant (both *P* > 0.05). At week 12, there was a small reduction in HbA1c level relative to baseline in saroglitazar group (−0.39%) when compared with an increase in HbA1c levels in fenofibrate group (4.28%). This difference was statistically significant (*P* = 0.023).

### Effect on liver enzyme

**Figure 2:**
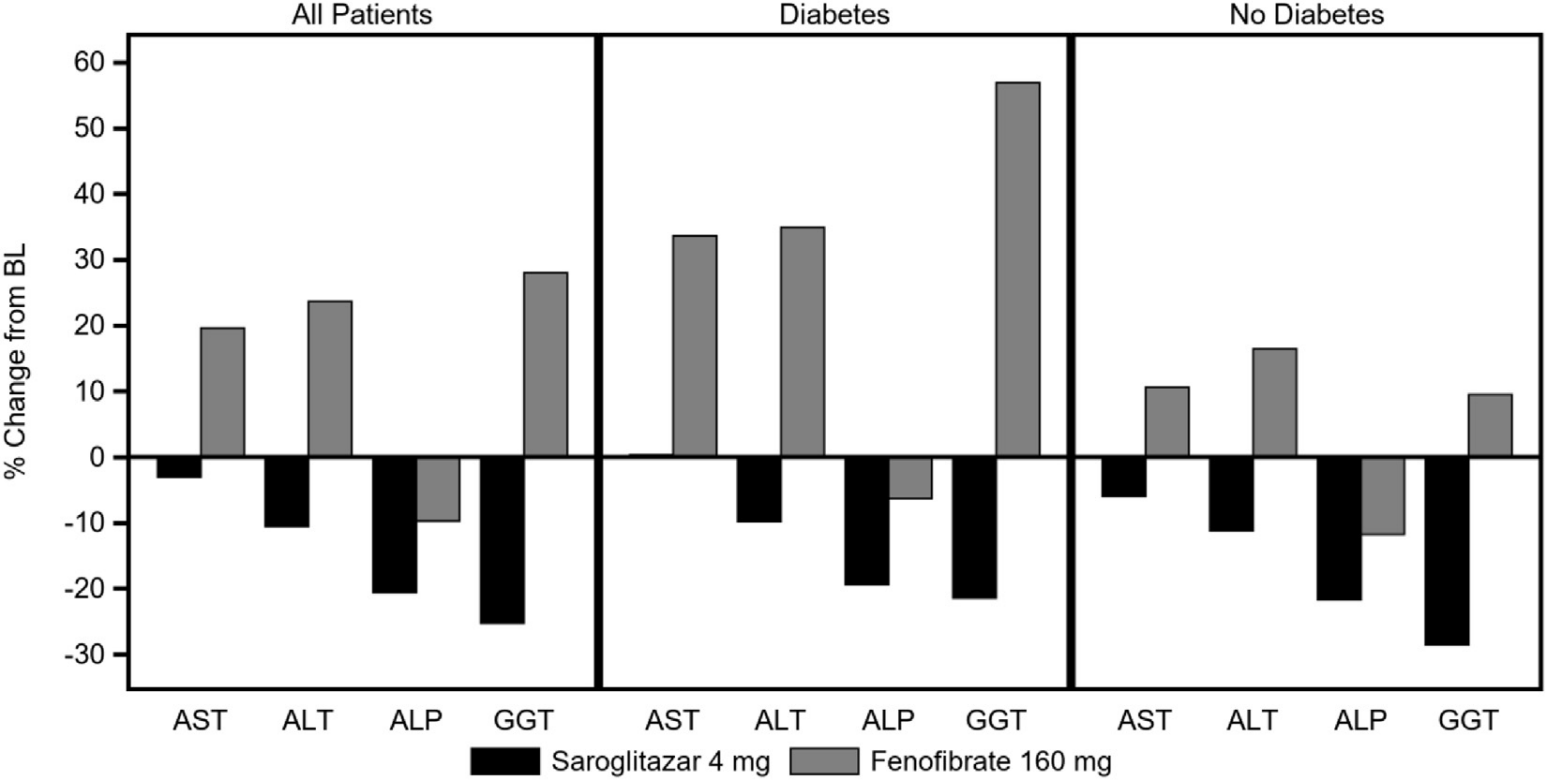
Percent change from baseline in liver enzyme parameters at week 12.

At week 12, the LS mean percent reduction in alanine transaminase, aspartate transaminase, and gamma-glutamyl transferase was −12.8%, −2.1%, and −24.7%, respectively, in the saroglitazar group when compared with a significant increase of 25.9% (*P* = 0.001), 18.6% (*P* = 0.018), and 27.4% (*P* = 0.005), respectively, in the fenofibrate group (Fig. 2). A significantly higher reduction in alkaline phosphatase at week 12 was observed in saroglitazar group (−21.3%) when compared with fenofibrate group (−9.1%, *P* = 0.003). No significant changes in liver stiffness and CAP at week 12 relative to baseline were observed in both the treatment groups (data not presented).

### Effect on subgroup analysis

**Figure 3:**
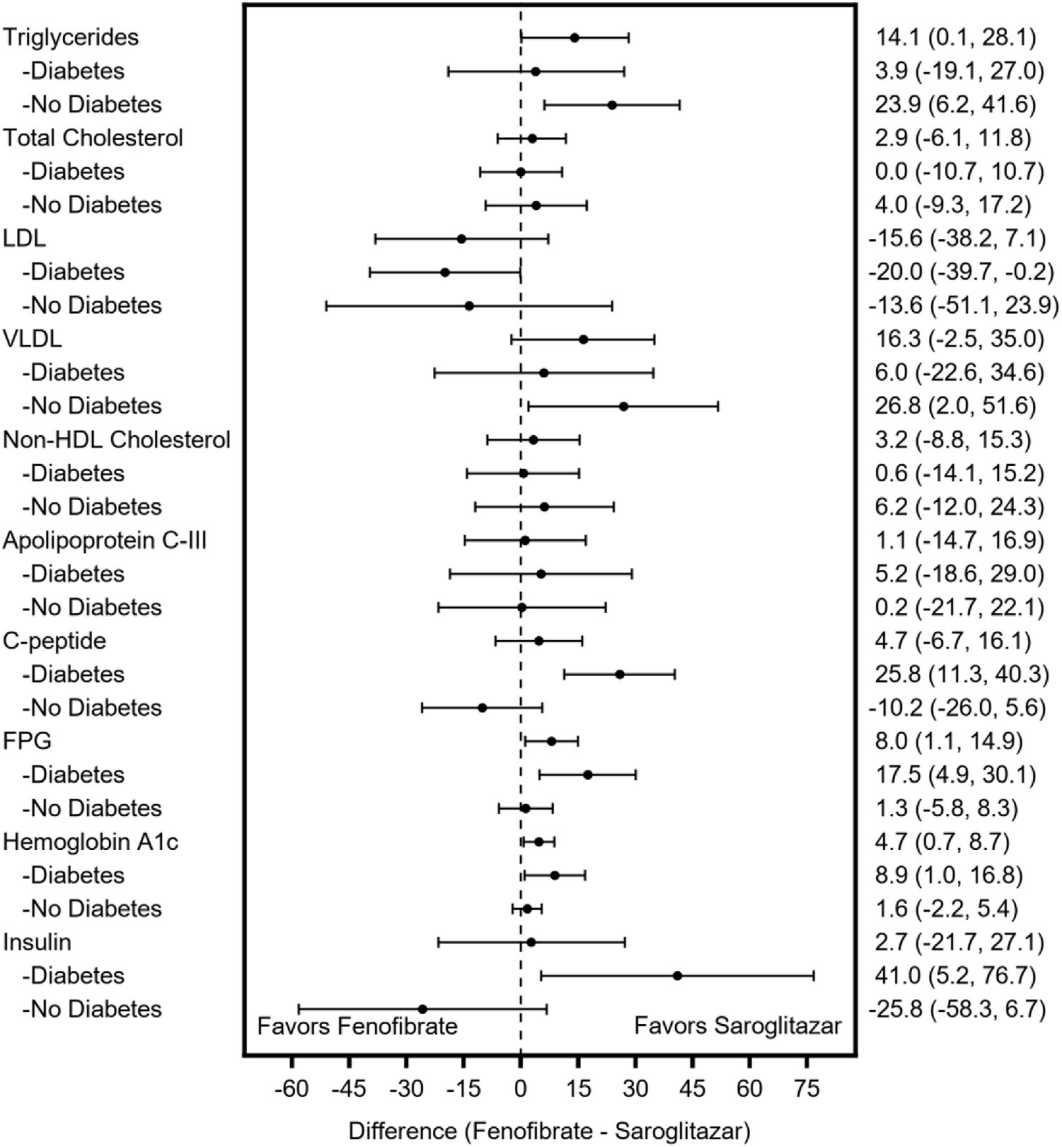
Forest plot showing the treatment differences (95% CI) in lipid and glucose control parameters.

Among participants without diabetes, saroglitazar had significant effect on mean treatment differences in reduction of TG (23.9% [95% CI: 6.2, 41.6], *P* = 0.009) and VLDL-C (26.8% [95% CI: 2.0%, 51.6%], *P* = 0.035) levels at week 12 when compared with fenofibrate (Fig. 3).

Among participants with diabetes, saroglitazar had significant effect on mean treatment differences in change from baseline in C-peptide (25.8% [95% CI: 11.3%], 40.3%), FPG (17.5% [95% CI: 4.9%, 30.1%], *P* = 0.008), HbA1c (8.9% [95% CI: 1%, 16.8%], *P* = 0.029), and insulin levels (41.0% [95% CI: 5.2%, 76.7%], *P* = 0.026) at week 12 when compared with fenofibrate (Fig. 3).

At week 12, participants with diabetes had a significantly higher increase in LDL-C levels in the saroglitazar group when compared with fenofibrate group (27.5% vs. 7.5%). This treatment difference was statistically significant (−20.0% [95% CI: −39.7%, −0.2%], *P* = 0.047).

### Safety profile

Overall, 37 treatment emergent AEs were reported in 24 patients; 13 (27.1%) patients in the saroglitazar group and 11 (23.9%) patients in the fenofibrate group. All reported AEs were mild to moderate in severity except one patient in the fenofibrate group, who experienced severe dyspepsia. The AEs reported were related to the study drug in one (2.1%) patient in the saroglitazar group and one (2.2%) patient in the fenofibrate group. No serious AEs were reported in both the treatment groups, and no patient discontinued the study because of AEs. No changes in creatinine levels in saroglitazar group (0.5%) were observed when compared with a significant increase in fenofibrate (10.7%) group (Table 3) at week 12. This difference was statistically significant (10.2% [95% CI: 1.5, 19.0], *P* = 0.023).

## Discussion

In this multicenter, randomized, and double-blind study, saroglitazar established noninferiority to fenofibrate with regard to percent reduction in TG levels at week 12 in patients with fasting TG level of 500–1,500 mg/dl. The percent reduction in TG levels at week 12 was 55.3% and 41.1% in saroglitazar and fenofibrate group, respectively. This study enrolled 35 (42.7%) participants with diabetes and 47 (57.3%) participants without diabetes in the PP population. In this study, the effect of saroglitazar was significantly higher in reducing the TG levels in participants with diabetes (−63.0%) when compared with participants without diabetes (−47.2%). Similar effect of saroglitazar in reducing TG levels was also observed in an earlier phase III study conducted in patients with hypertriglyceridemia (>200 and <500 mg/dl) with type 2 diabetes mellitus who were not controlled with statin therapy (PRESS VI (13)).

Overall, improvement in TC, non-HDL-C, VLDL-C, HDL-C, and apolipoprotein C-III levels at week 12 relative to baseline was observed in both the treatment groups. A significantly higher reduction in VLDL-C was observed in favor of saroglitazar compared with fenofibrate in participants without diabetes. Previous studies in patients with TG <500 mg/dl have shown decreased LDL-C levels with saroglitazar treatment (13, 14, 15). In this study, a significant increase in LDL-C was observed in both saroglitazar and fenofibrate groups. The observed increases in LDL-C in both cohorts were not unexpected. Our results correlate with previous studies of TG-lowering treatments in patients with TG >500 mg/dl where reduction in TG levels was associated with increase in LDL-C (16, 17, 18, 19, 20). This is likely caused by increasing intravascular lipolysis of VLDL-C through LPL, with resultant accumulation of nonatherogenic form of LDL-C (21).

These findings were broadly in line with the lipid profile changes seen in the previous phase 3 studies of this development program (PRESS V (15) and PRESS VI (13)). In PRESS V, which compared saroglitazar with pioglitazone, 122 patients with diabetic dyslipidemia (TG >200–400 mg/dl) had a decrease in TG at week 24 (45% and 15.5%, respectively), which was statistically significant. Saroglitazar also significantly decreased LDL-C, VLDL-C, and TC compared with pioglitazone and numerically increased HDL-C at week 24 (15).

There was a small decrease in FPG levels (6%) across postbaseline time points with saroglitazar, consistent with the change in HbA1c, especially in participants with diabetes. At week 12, there was a significant increase in HbA1c with fenofibrate of 4.3% and a very small decrease with saroglitazar of 0.4% (*P* < 0.05). This treatment difference is more pronounced in participants with diabetes, which signifies additional effect of saroglitazar on glycemic control via its PPAR-γ agonist action. Adiponectin is an adipose-specific secretory protein and acts as an antidiabetic and antiatherosclerotic molecule. Furthermore, a number of clinical trials showed that subjects with high levels of circulating adiponectin tend to be protected against type 2 diabetes and myocardial infarction (22). Adiponectin levels were increased with both treatments, and there was a statistically significant treatment difference (*P* < 0.0001) owing to the large increase after saroglitazar (49.3%) and the modest increase after fenofibrate (6.2%). This effect of saroglitazar corresponds to additional PPARγ-mediated upregulation of adiponectin at the transcription level with resultant increase in adiponectin levels in humans. Compared to fenofibrate, saroglitazar treatment showed significant reduction in fasting insulin and C-peptide in participants with diabetes, indicating improvement in insulin resistance. Contrary to fenofibrate treatment, which showed elevation in liver serum transaminases (alanine transaminase and aspartate transaminase) and gamma-glutamyl transferase, saroglitazar showed reduction in liver parameters. Similar to prior studies (23), this study also showed increased creatinine in fenofibrate treatment arm. However, no change in creatinine was observed with saroglitazar.

Overall, this study demonstrated the noninferiority of saroglitazar 4 mg to fenofibrate 160 mg in reducing TG levels at week 12 in patients with moderate to severe hypertriglyceridemia. A significantly higher percentage of patients in the saroglitazar 4 mg achieved TG level of <500 mg/dl at week 12 when compared with fenofibrate 160 mg. Based upon a review of AEs, vital signs, laboratory assessments, electrocardiogram, and physical examination assessments, saroglitazar 4 mg was generally well tolerated when administered orally for 12 weeks in adult patients with moderate or severe hypertriglyceridemia.

## Data availability

The deidentified individual patient data would be available along with the protocol and the statistical analysis plan immediately following the publications for anyone who wishes to access the data by contacting the corresponding author.

## Supplemental data

This article contains supplemental data.

## Conflict of interest

The authors declare that they have no conflicts of interest with the contents of this article. D. P. was from Zydus Therapeutics, Inc.; F. S. was from Zydus Worldwide DMCC; R. R.-G. and J. G. G. are from the School of Medicine and University Hospital, Autonomous University of Nuevo León, Mexico. P. C.-L. is from the Avant Sante Research Center. Clinical trial registration: SARO.17.001.02.PROT.
