# Supplementary figures and images for: Saroglitazar is noninferior to fenofibrate in reducing triglyceride levels in hypertriglyceridemic patients in a randomized clinical trial
Source: J Lipid Res. 2022 May 21;63(7):100233. doi: 10.1016/j.jlr.2022.100233 (PMC9240860; doi:10.1016/j.jlr.2022.100233)

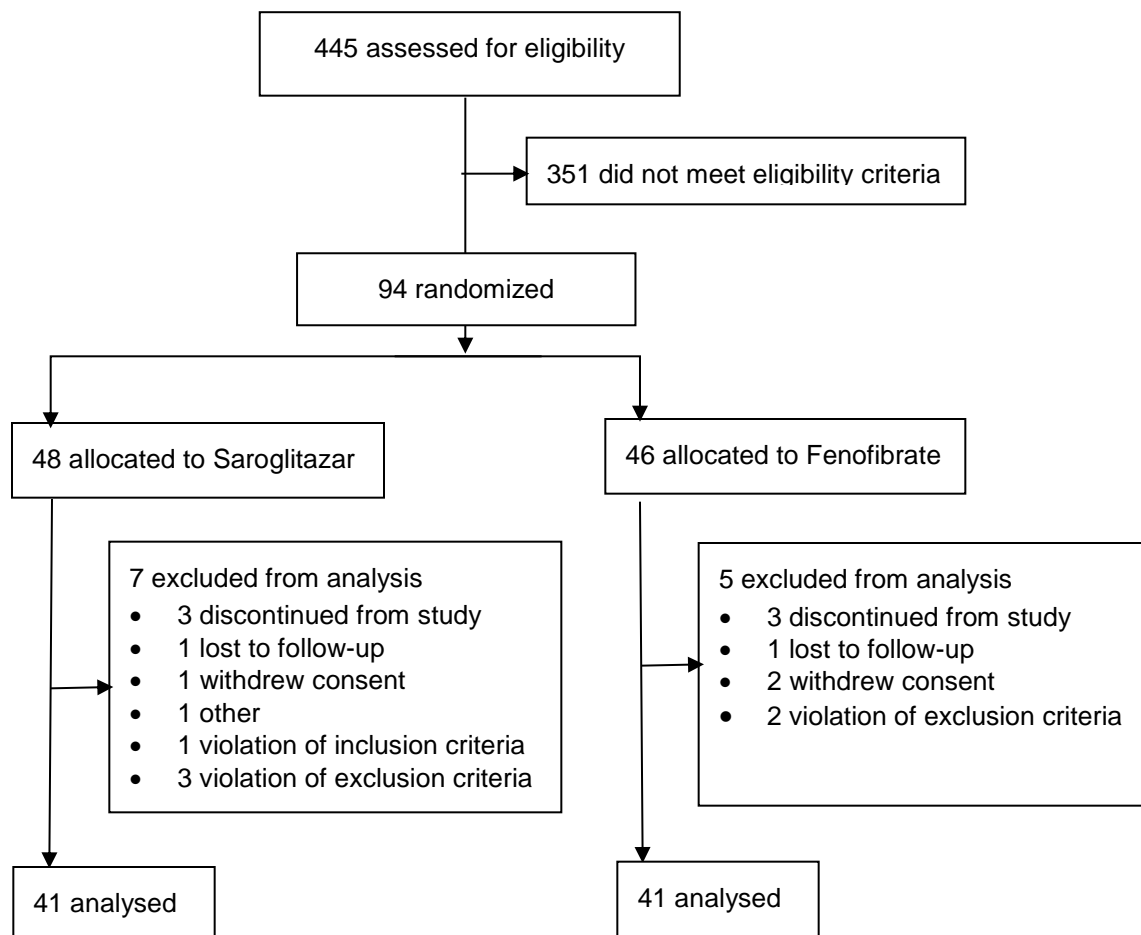

Supplement: Supplementary file 1 [file mmc1.pdf]
